# Preconceptional Risk Factors Associated With Gestational Diabetes Mellitus and Pregnancy Outcomes: A Case-Control Study

**DOI:** 10.7759/cureus.116764

**Published:** 2026-09-23

**Authors:** Dharitri Swain, Priyadarshini T., Tania Mishra, Subhasmita Das, Subhoshree Das, Sutapa Das, Tamanna Koley, Triparna Halder

**Affiliations:** 1 Obstetrics and Gynecological Nursing, All India Institute of Medical Sciences, Bhubaneswar, IND; 2 College of Nursing, All India Institute of Medical Sciences, Bhubaneswar, IND

**Keywords:** gestational diabetes mellitus, lifestyle factors and gdm, preconception counselling, pregnancy-related outcomes, risk factors of gdm

## Abstract

Introduction: Gestational diabetes mellitus (GDM) is a major endocrine problem complicating pregnancy worldwide, and pregnancies complicated by GDM are associated with adverse birth outcomes. This study aimed to identify preconceptional risk factors and birth outcomes associated with GDM.

Methodology: A hospital-based case-control study was conducted among antenatal mothers seeking routine antenatal care at selected maternity hospitals in Khurdha district, Odisha, India. The study comprised 143 women with GDM and 143 gestational age-matched controls. Data were collected using a structured questionnaire and interviews. Data were entered and analyzed using SPSS, version 21 (IBM Corp., Armonk, NY).

Results: The point prevalence of GDM in the study population was 9.9%. The preconceptional risk factors, such as preconception body mass index (BMI) (odds ratio (OR) = 3.01, 95% confidence interval (CI): 1.78-5.09, *P* < 0.001), sedentary lifestyle (OR = 2.79, 95% CI: 1.33-5.88, *P* = 0.00), previous history of diabetes (OR = 3.72, 95% CI: 1.33-10.39, *P* = 0.00), family history of diabetes (OR = 1.94, 95% CI: 1.16-3.24, *P* = 0.01), and domestic violence (OR = 2.14, 95% CI: 1.13-4.06, *P* = 0.02) had significantly higher odds of developing GDM compared with non-GDM women. In relation to the obstetrical risk factors, early age at menarche (≤12 years) (OR = 2.79, 95% CI: 1.72-4.54, *P* < 0.001), previous history of GDM (OR = 2.42, 95% CI: 1.06-5.52, *P* = 0.03), primipara mothers (OR = 2.21, 95% CI: 1.38-3.56, *P* = 0.001), previous history of macrosomia (OR = 16.6, 95% CI: 2.16-127.76, *P* < 0.001), history of preterm delivery (OR = 2.78, 95% CI: 1.35-5.69, *P* = 0.004), and history of using steroid medications (OR = 2.42, 95% CI: 1.06-5.52, *P* = 0.03) increased the likelihood of developing GDM in the subsequent pregnancy. Furthermore, GDM was significantly associated with antepartum depression and adverse intrapartum and neonatal outcomes. Women with GDM had significantly higher odds of developing maternal depression (OR = 2.55, 95% CI: 1.45-4.50, *P* = 0.001), cesarean delivery (OR = 2.92, 95% CI: 1.79-4.76, *P* < 0.001), and preterm delivery (OR = 1.94, 95% CI: 1.19-3.15, *P* = 0.00), and the need for induction of labor was significantly higher among women with GDM (OR = 4.11, 95% CI: 2.47-6.85, *P* < 0.001) compared with non-GDM women. With respect to neonatal outcomes, mothers with GDM had significantly higher odds of developing neonatal hyperbilirubinemia (OR = 7.05, 95% CI: 1.56-31.84, *P* = 0.01), neonatal hypoglycemia (OR = 14.00, 95% CI: 4.18-46.85, *P* = 0.001), giving birth to a macrosomia baby (OR = 32.97, 95% CI: 4.30-4235.35, *P* = 0.01), and being admitted to the neonatal intensive care unit (NICU) (OR = 5.68, 95% CI: 3.34-9.64, *P* = 0.001).

Conclusions: It was shown that maternal preconception lifestyle risk factors and poor obstetric factors were associated with GDM in the subsequent pregnancy and with unfavorable pregnancy-related outcomes. Preconception counseling, along with need-based appropriate management, is highly important for addressing modifiable risk factors and reducing unfavorable birth outcomes associated with GDM.

## Introduction

Gestational diabetes mellitus (GDM) is an important global public health concern and one of the most common metabolic complications of pregnancy [1]. According to the World Health Organization (WHO), GDM is defined as hyperglycemia first detected during pregnancy, which may be diagnosed at any time during pregnancy when the specified diagnostic thresholds are met [2].The development of GDM is primarily associated with increased insulin resistance during pregnancy, particularly as gestation advances, when placental hormones contribute to impaired glucose metabolism and hyperglycemia [3]. The prevalence of GDM varies substantially across populations due to differences in maternal characteristics, including age, socioeconomic status, ethnicity, body composition, and lifestyle, as well as variations in screening strategies and diagnostic criteria [4].

Globally, hyperglycemia in pregnancy represents a substantial public health burden, with approximately 21 million pregnancies affected annually, and a disproportionately high burden occurring in low- and middle-income countries. South and Southeast Asia, including India, have reported high prevalence rates of GDM [5,6]. The prevalence of GDM in Asian populations is expected to increase further because of increasing maternal age, rising prevalence of overweight and obesity, sedentary lifestyles, and changing dietary patterns [7].

India carries a substantial burden of GDM. A nationally representative analysis estimated that approximately 5-8 million pregnant women may be affected by GDM annually [8]. However, considerable variation in prevalence has been reported across regions of India, with estimates ranging from approximately 3.8% to 17.9%, reflecting differences in population characteristics, socioeconomic conditions, dietary and lifestyle practices, screening approaches, and diagnostic criteria [9-12]. In Odisha, the prevalence of GDM has been reported to be approximately 9.89% [12], indicating a substantial regional burden and highlighting the need for further investigation into factors associated with GDM in this population.

The development of GDM is multifactorial and is influenced by a combination of preconception, sociodemographic, lifestyle, medical, and obstetric factors. Several maternal and preconception characteristics have consistently been associated with an increased risk of GDM, including advanced maternal age, overweight or obesity, higher pre-pregnancy body mass index (BMI), previous history of GDM, previous delivery of a macrosomic infant, and family history of diabetes [11-16]. Other reported obstetric factors include multiparity, excessive gestational weight gain, short maternal stature, polycystic ovary syndrome (PCOS), elevated maternal iron stores, previous cesarean delivery, adverse previous pregnancy outcomes such as spontaneous abortion, fetal loss, or congenital anomalies, and hypertension [16-19]. Although these factors have been identified in previous studies, their prevalence and strength of association with GDM may vary across populations and geographical settings. Differences in maternal nutritional status, dietary practices, physical activity, socioeconomic circumstances, reproductive characteristics, and access to healthcare may influence the distribution and contribution of these factors. Therefore, identification of population-specific factors associated with GDM is important for improving risk assessment and developing appropriate preventive strategies, particularly in settings with a high burden of GDM.

GDM is also associated with an increased risk of adverse maternal, obstetric, and neonatal outcomes. Women with GDM have higher risks of hypertensive disorders of pregnancy, pre-eclampsia, induction of labor, cesarean delivery, polyhydramnios, premature rupture of membranes, postpartum hemorrhage, and preterm birth. For newborns, GDM is associated with increased risks of macrosomia, neonatal hypoglycemia, prematurity, hyperbilirubinemia, birth injury, and other perinatal complications [17-19]. These adverse outcomes emphasize the importance of identifying women at increased risk of GDM and ensuring timely screening, diagnosis, appropriate management, and monitoring during pregnancy.

In India, the prevention and management of GDM remain challenging due to inadequate awareness, sociocultural and dietary practices, limited access to screening and diagnostic services, and variations in screening, diagnostic, and management practices [20,21].Identification of women with important preconception risk factors provides an opportunity for early counseling, lifestyle modification, timely screening, and appropriate management. However, region-specific evidence regarding the contribution of socioeconomic, lifestyle, obstetric, and medical characteristics to GDM remains limited, particularly in Odisha.

Therefore, the present study was undertaken to determine the prevalence of GDM, identify preconception socioeconomic, lifestyle, obstetric, and medical factors associated with GDM, and evaluate birth outcomes among women attending public healthcare facilities. Identifying these factors may facilitate early recognition of women at increased risk and contribute to the development of targeted preventive and clinical strategies for reducing GDM-related maternal and neonatal complications.

## Materials and methods

Study design and setting

This case-control study design identified risk factors and birth outcomes associated with GDM among mothers compared with non-GDM mothers attending the study centers' antenatal clinics. The study was conducted for six months at selected public maternity healthcare centers in Khurda district, Odisha, Southeastern India.

Eligibility of participants

The cases were diagnosed with GDM at 24-28 weeks of gestation using a one-step strategy (75-g, two-hour oral glucose tolerance test), according to the criteria of the American Diabetes Association (ADA)/International Association of Diabetes and Pregnancy Study Groups (IADPSG). Eligibility criteria for the cases were women with GDM aged 18 to 40 years, a gestational age (GA) of 24 to 28 weeks, consent, and willingness to participate in this study during their antenatal visits. Healthy pregnant women with normal glucose tolerance test results, who were in a similar GA group, were selected from the same hospital during their antenatal visits and served as the control group. Cases and controls were matched on GA to ensure comparability with respect to the physiological progression of pregnancy. As insulin resistance and placental hormone levels increase with advancing gestation, GA is a strong determinant of glucose metabolism and GDM diagnosis. Matching on GA minimized potential confounding and enhanced the study's internal validity by ensuring comparability between groups with respect to physiological changes associated with advancing gestation.

Women with preconception diabetes (type 1, type 2, or both types of diabetes), recurrent miscarriages, multiple gestations, known chronic illnesses such as hypertension, chronic renal disease, collagen or vascular diseases, endocrine disorders, chronic hepatic diseases, and current use of corticosteroids were excluded from the study. The selection of participants is presented in Figure 1.

**Figure 1:**
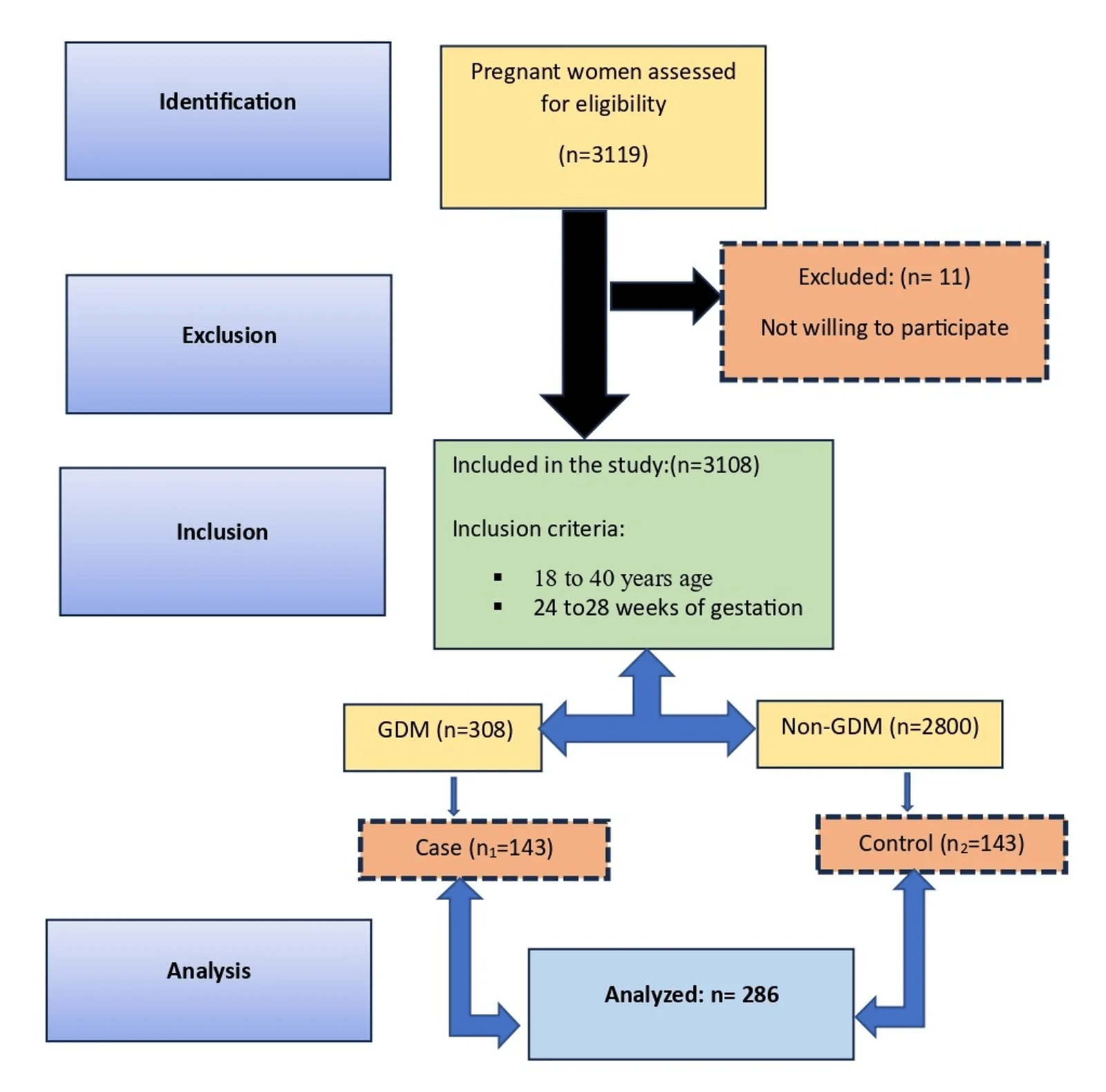
Flow diagram of study participant selection. Image created by the author using Microsoft Word (Microsoft Corp., Redmond, WA). GDN, gestational diabetes mellitus

Sample size and sample recruitment

The sample size was calculated using a standardized statistical formula for a case-control study (r+1/r SD2(Zβ + Zα/2) 2/d2), with a 5% significance level and 80% power, based on a previous Indian study [20]. The total sample size was estimated at 143 participants in each of the case and control groups.

The participant selection process is illustrated in Figure 1. A total of 3,119 pregnant women were assessed for eligibility, of whom 11 were excluded due to unwillingness to participate. The remaining 3,108 women met the eligibility criteria: being 18-40 years of age and having a GA of 24-28 weeks. Among the 3,108 eligible women, 308 were diagnosed with GDM and constituted the case population. Of these, 143 women with GDM were selected using simple random sampling with computer-generated random numbers. An equal number of women without GDM (*n* = 143) were selected from the eligible non-GDM population using the same method. Thus, a 1:1 ratio of cases to controls was maintained, resulting in a total sample of 286 participants (143 cases and 143 controls).

Ethical approval and consent to participate

Ethical approval was obtained from the Institutional Ethics Committee and the concerned hospital authority prior to data collection. The investigator explained the purpose of the research and the interview duration to all participants by providing a patient information sheet (PIS). Written informed consent was obtained from all participants for their voluntary participation in the study, and they had the right to withdraw at any time during the data collection period, as outlined in the patient information consent form (PICF). It was assured that confidentiality would be maintained and that the data would not be used for any purpose other than research.

Data collection tool and procedures

The data were collected through face-to-face interviews using a set of structured questionnaires, with each participant interviewed separately for 10-15 minutes. Anonymity and confidentiality were assured during the data collection process. A detailed history was taken, and a physical examination was performed for all pregnant women. Preconception weight was obtained by self-report at enrolment, and height was measured using a standardized stadiometer. When women did not recall their pre-pregnancy weight, the earliest measured maternal weight in the first trimester from existing antenatal records was used as the pre-pregnancy weight after subtracting an estimated first-trimester weight gain of 0.5-2.0 kg. BMI was calculated by dividing weight in kilograms by height in meters squared and categorized according to standard cutoffs (underweight <18.5 kg/m^2^, normal 18.5-24.9 kg/m^2^, overweight 25-29.9 kg/m^2^, obese ≥30 kg/m^2^).

A structured and standardized tool was administered to assess sociodemographic factors and preconceptional risk factors. Baseline characteristics were assessed using a semi-structured demographic and prenatal proforma. Sociodemographic factors included age, maternal and paternal education levels, place of residence, occupation, and socioeconomic status (a monthly income below ₹10,000 in rural areas and ₹15,000 in urban areas was considered the BPL category). The preconceptional risk factors, such as preconception health, lifestyle factors, preconception comorbidities, and previous obstetrical factors, were included in the study. Preconceptional health included BMI, menstrual cycle pattern, contraceptive use, maternal stress, domestic violence, etc. Lifestyle factors included physical activity, dietary pattern, sleep pattern, substance abuse, use of medications, etc.

We used self-report interview methods to assess preconceptional physical activity as sedentary or active in terms of the type of activity (walking/cycling/yoga/running/any other), frequency (days per week), and duration (minutes per day). Finally, physical activity levels were classified according to WHO recommendations as sedentary (<150 min/week) and active (≥150 min/week). The self-report interview method was also used to assess dietary pattern (vegetarian or non-vegetarian), dietary components (high-carbohydrate or normal diet), sleeping pattern (more or less than seven hours), history of substance abuse, menstrual cycle pattern, contraceptive use, and domestic violence.

Maternal depression during pregnancy was assessed using the Edinburgh Postnatal Depression Scale (EPDS), Odia version [22,23]. The EPDS is a 10-item self-report questionnaire, with each item scored on a 4-point scale ranging from 0 to 3 based on symptom severity. The total score ranges from 0 to 30, with higher scores indicating greater depressive symptoms. A cutoff score of ≥12 was used to identify mothers with probable antepartum depression [24]. Antenatal depression was assessed during the enrollment period in the second and third trimesters of pregnancy.

Preconceptional comorbidities included previous medical conditions such as diabetes, hypertension, thyroid disorders, PCOS, anemia, etc. Obstetrical factors included previous pregnancy complications, present pregnancy complications, gravidity, parity, previous mode of delivery, pregnancy and childbirth history, exercise during pregnancy, antenatal visits, and preference of delivery mode. Data were obtained from existing antenatal records and through face-to-face interviews by the investigator. A structured questionnaire was administered to assess birth outcomes associated with GDM, and data were recorded from the postnatal record.

The content validity and reliability of the tool were established prior to administration, and the same investigator was assigned to interview all participants to minimize interpretation bias. The overall content validity of the structured tool was appropriate, with a content validity ratio (CVR) of 0.58 across five experts and a content validity index (CVI) of 0.89. The internal consistency reliability of the scale items was satisfactory, with a Cronbach’s alpha coefficient of 0.845. The tool was translated into the regional language (Odia) to facilitate participant understanding, and its language validity was assessed by a language expert.

Outcome assessment

The pregnancy outcomes included preeclampsia, oligohydramnios, and polyhydramnios. Oligohydramnios was diagnosed when the amniotic fluid index (AFI) was ≤5 cm or the single deepest pocket (SDP) was <2 cm, while polyhydramnios was diagnosed when the AFI was ≥24 cm or the SDP was ≥8 cm based on ultrasound measurements. The labor/intrapartum outcomes included mode of delivery, emergency cesarean section (CS), premature rupture of membranes (PROM) (rupture of the membranes more than one hour before the onset of labor), and preterm delivery (delivery before 37 weeks of gestation). Neonatal outcomes included fetal death, macrosomia (birth weight at delivery >4 kg), intrauterine growth restriction (IUGR) (growth <3rd percentile for GA), congenital malformation (any structural or functional abnormality present at birth and documented in the newborn's medical record), neonatal hypoglycemia (blood glucose concentration <45 mg/dL), neonatal hyperbilirubinemia (according to the American Academy of Pediatrics (AAP) guidelines and documented in the newborn's medical record), and neonatal intensive care unit (NICU) admission (admission to the NICU requiring specialized neonatal care). The selected outcome measures were recorded from the postnatal records after delivery.

Statistical analysis

The collected data were coded and entered into an Excel spreadsheet, cleaned, and checked for missing values. SPSS Statistics, version 20 (IBM Corp., Armonk, NY) was used to analyze the data. The baseline characteristics and clinical parameters were expressed as frequencies, means, and standard deviations. Unadjusted odds ratios (ORs) were used to identify factors associated with GDM and associated pregnancy-related outcomes. The ORs and Fisher’s exact *P*-values were computed for small or imbalanced cell counts. Because of sparse data, Firth’s penalized logistic regression was applied to outcomes with zero cells. Cases and controls were matched on GA; hence, GA was not included as an independent variable in the regression models. 

## Results

Sample characteristics

The demographic and reproductive characteristics of the parturients are summarized in Table 1. A total of 286 parturients (143 with GDM and 143 without GDM) were included in the final analysis. There was no statistically significant difference in the mean GA at the time of GDM assessment between the two groups (*P* = 0.61), indicating good comparability of cases and controls with respect to GA. The mean age and marriage age of the participants were 23.09 ± 2.326 and 23.9 ± 2.603, respectively. The majority of participants (222, 77.6%) were aged 18-30 years; all were married, and the majority (266, 93%) were married at ages 15-30 years. Most of the participants (246, 86%) were housewives; 138 (48.2%) had completed their higher-level education; the majority of them (254, 88.81%) belonged to the above poverty line (APL) group; and more than half of the subjects (167, 58.39%) were from the urban population. The mean BMI was 24.43 ± 2.21 kg/m^2^. 

**Table 1:** Demographic characteristics of participants (n = 286). GDM, gestational diabetes mellitus; APL, above poverty line; BPL, below poverty line

Characteristics	Total (*n *= 286)	GDM (*n* = 143)	Non-GDM (*n *= 143)	*P*-value
	Mean ± SD or *n* (%)	Mean ± SD or *n* (%)	Mean ± SD or *n* (%)	
Gestational age at enrolment (weeks)	26.09 ± 1.16	26.12 ± 1.1	26.05 ± 1.22	0.61
Age (years)	23.09 ± 2.32	23.33 ± 2.24	22.85 ± 2.40	0.08
Age at marriage (years)	23.90 ± 2.60	24.60 ± 2.35	23.2 ± 2.85	< 0.001*
Height (cm)	157.54 ± 5.92	157.58 ± 6.05	157.50 ± 5.80	0.91
Weight (kg)	60.56 ± 5.96	61.53 ± 6.05	59.59 ± 5.88	0.00
Body mass index (BMI)	24.43 ± 2.21	24.82 ± 2.1	24.05 ± 2.32	0.004
Age (years)				0.52
<18	03 (1.10)	01 (0.70)	02 (1.40)	
18-29	222 (77.62)	108 (75.50)	114 (79.70)	
≥30	61 (21.32)	34 (23.80)	27 (18.90)	
Age at marriage (years)				0.63
<18	17 (5.90)	07 (4.90)	10 (7)	
18-30	266 (93)	135 (94.40)	131 (91.60)	
31-35	03 (1.10)	01 (0.70)	02 (1.40)	
Marital status				
Married	286 (100%)	143 (100%)	143 (100%)	-
Education				0.85
No formal education	08 (02.79)	3 (2.10)	5 (3.50)	
Primary school	34 (11.88)	16 (11.20)	18 (12.60)	
Secondary school	106 (37.06)	55 (38.40)	51 (35.60)	
College or university	138 (48.25)	69 (48.30)	69 (48.30)	
Current occupation of women				0.38
Housewife	246 (86.01)	128 (89.50)	118 (82.50)	
Govt. job	23 (08.04)	9 (6.30)	14 (9.80)	
Private job	05 (01.74)	2 (1.40)	3 (2.10)	
Others	12 (04.19)	4 (2.80)	8 (5.60)	
Current occupation of husband				0.77
Unemployment	09 (03.14)	3 (2.10)	6 (4.20)	
Govt. job	89 (31.11)	46 (32.20)	43 (30.10)	
Private job	89 (31.11)	45 (31.40)	44 (30.70)	
Others	99 (34.61)	49 (34.30)	50 (35.50)	
Economic status				0.06
APL	254 (88.81)	122 (85.30)	132 (92.30)	
BPL	32 (11.18)	21 (14.70)	11 (7.70)	
Habitat				0.55
Urban habitat	167 (58.39)	86 (60.10)	81 (56.60)	
Rural habitat	119 (41.60)	57 (39.90)	62 (43.30)	

Point prevalence and risk factors of GDM

Of the 3,108 study participants, 308 women were diagnosed with GDM using the *one-step strategy* (75-g, two-hour oral glucose tolerance test), yielding a point prevalence of 9.9% over a six-month data collection period. A total of 143 women with GDM and 143 without GDM were included in the analysis, and unadjusted ORs were used to identify factors associated with GDM and associated pregnancy-related outcomes.

Preconceptional risk factors

No significant association was established between GDM and sociodemographic factors (Table 2). In contrast, preconceptional risk factors such as preconception BMI (OR = 3.01, 95% CI: 1.78-5.09, *P* < 0.001), sedentary lifestyle (OR = 2.79, 95% CI: 1.33-5.88, *P* = 0.00), previous history of diabetes (OR = 3.72, 95% CI: 1.33-10.39, *P* = 0.00), family history of diabetes (OR = 1.94, 95% CI: 1.16-3.24, *P* = 0.01), and domestic violence (OR = 2.14, 95% CI: 1.13-4.06, *P* = 0.02) had significantly higher odds of developing GDM compared with non-GDM women. No significant difference was found in the odds of having GDM between individuals related to diet pattern, history of substance abuse, sleeping pattern, previous history of PCOS, and use of oral contraceptive pills (OCPs) (Table 3).

**Table 2:** Sociodemographic risk factors associated with gestational diabetes mellitus (GDM) (n = 286). Fisher’s exact *P*-values were computed for small or imbalanced cell counts. GDM, gestational diabetes mellitus; APL, above poverty line; BPL, below poverty line; OR, odds ratio; CI, confidence interval

Variables	GDM (*n* = 143), *n* (%)	Non-GDM (*n* = 143), *n* (%)	OR (95% CI)	*P*-value
Age (years)				
<30	109 (76.20)	116 (81.10)	0.75 (0.42-1.32)	0.31
>=30	34 (23.70)	27 (18.90)	1	-
Age at marriage				
<30	142 (99.30)	141 (98.60)	2.01 (0.18-22.47)	1.00
>=30	1 (0.70)	2 (1.40)	1	-
Education				
No formal education	3 (2.10)	5 (3.50)	0.59 (0.14-2.52)	0.72
Formal education	140 (97.90)	138 (96.50)	1	
Occupation				
Unemployed	128 (89.50)	118 (82.50)	1.80 (0.90-3.59)	0.09
Employed	15 (10.50)	25 (17.40)	-	-
Occupation of husband				
Unemployed	3 (2.10)	6 (4.20)	0.49 (0.12-2.00)	0.50
Employed	140 (97.90)	137 (95.80)	1	-
Economic status				
APL	122 (85.30)	132 (92.30)	0.48 (0.22-1.04)	0.06
BPL	21 (14.60)	11 (7.60)	1	-
Habitat				
Urban habitat	86 (60.10)	81 (56.60)	1.15 (0.72-1.85)	0.55
Rural habitat	57 (39.90)	62 (43.30)	1	-

**Table 3:** Preconception factors associated with gestational diabetes mellitus (GDM) (n = 286). *Statistically significant (*P* < 0.05). Fisher's exact *P*-values were computed for small or imbalanced cell counts. GDM, gestational diabetes mellitus; PCOS, polycystic ovary syndrome; OCP, oral contraceptive pill; OR, odds ratio; CI, confidence interval

Variables	GDM (*n* = 143), *n* (%)	Non-GDM (*n* = 143), *n* (%)	OR (95% CI)	*P*-value
Preconception BMI				
≥25	62 (43.36)	29 (20.28)	3.01 (1.78-5.09)	<0.001*
<25	81 (56.60)	114 (79.70)	1	-
Physical activity				
Sedentary	27 (18.88)	11 (07.69)	2.79 (1.33–5.88)	0.00*
Active	116 (81.12)	132 (92.31)	1	-
Diet pattern				
Non-veg	89 (62.20)	101 (70.60)	0.69 (0.42-1.12)	0.13
Veg	54 (37.70)	42 (29.30)	-	-
Dietary component				
High carbohydrate	29 (20.20)	34 (23.70)	0.82 (0.47-1.43)	0.47
Normal diet	114 (79.70)	109 (76.20)	1	
Substance use (corrected with Firth regression)				
Yes	1 (0.70)	0 (0)	3.02 (0.12–74.10)	0.54
No	142 (99.30)	143 (100)		
Hours of sleep				
<7 hours	44 (30.70)	42 (29.30)	1.07 (0.64-1.77)	0.79
>7 hours	99 (69.20)	101 (70.60)	1	-
Pattern of menstrual cycle				
Regular	105 (73.40)	100 (69.90)	1.19 (0.71-1.99)	0.51
Irregular	38 (26.50)	43 (30)	1	-
Family history of diabetes				
Yes	54 (37.76))	34 (23.78)	1.95 (1.17-3.25)	0.01*
No	89 (62.20)	109 (76.20)	1	-
Previous history of diabetes				
Yes	17 (11.89)	05 (3.50%)	3.72 (1.33-10.39)	0.00*
No	126 (88.10)	138 (96.50)	1	-
Previous history of PCOS				
Yes	14 (9.70)	20 (13.90)	0.67 (0.32–1.38)	0.27
No	129 (90.20)	123 (86)	1	-
Use of OCP				
Yes	22 (15.30)	27 (18.80)	0.78 (0.42-1.45)	0.43
No	121 (84.60)	116 (81.10)	1	-
Domestic violence				
Yes	32 (22.38))	17 (11.89)	2.14 (1.13-4.06)	0.02*
No	111 (77.62)	126 (88.11)	1	-

Obstetrical risk factors of GDM

In relation to the obstetrical risk factors, early age at menarche (≤12 years) (OR = 2.79, 95% CI: 1.72-4.54, *P* < 0.001), previous history of GDM (OR = 2.42, 95% CI: 1.06-5.52, *P* = 0.03), primipara mothers (OR = 2.22, 95% CI: 1.38-3.56, *P* = 0.001), previous history of macrosomia (OR = 16.6, 95% CI: 2.17-127.76, *P* < 0.001), history of preterm delivery (OR = 2.78, 95% CI: 1.35-5.69, *P* = 0.004), and history of using steroid medications (OR = 2.42, 95% CI: 1.06-5.52, *P* = 0.03) increased the likelihood of developing GDM in the subsequent pregnancy. Furthermore, maternal depression during pregnancy was found to be significantly associated with GDM (OR = 2.55, 95% CI: 1.45-4.50, *P* = 0.001) (Table 4).

**Table 4:** Obstetric risk factors associated with gestational diabetes mellitus (GDM) (n = 286). *Statistically significant (*P* < 0.05). Fisher's exact *P*-values were computed for small or imbalanced cell counts. GDM, gestational diabetes mellitus; EPDS, Edinburgh Postnatal Depression Scale; OR, odds ratio; CI, confidence interval

Variables	GDM (*n* = 143), *n* (%)	Non-GDM (*n* = 143), *n* (%)	OR (95% CI)	*P*-value
Age at menarche (years)				
≤12	78 (54.54)	43 (30.07)	2.79 (1.72-4.54)	<0.001*
>12	65 (45.45)	100 (69.93)	1	
Previous history of GDM				
Yes	20 (13.98)	09 (6.29)	2.42 (1.06-5.52)	0.03*
No	123 (86.01)	134 (93.70)	1	-
Gravida				
Primigravida	71 (49.65)	79 (55.24)	0.79 (0.50-1.27)	0.34
Multigravida	72 (50.34)	64 (44.75)	1	-
Parity				
Primipara	81 (56.64)	53 (37.06)	2.22 (1.38-3.56)	0.001*
Multipara	62 (43.35)	90 (62.93)	1	-
History of miscarriage				
Yes	13 (9.09)	17 (11.88)	0.74 (0.34-1.59)	0.44
No	130 (90.90)	126 (88.11)	1	-
Previous history of macrosomia (baby's birth weight > 4 kg)				
Yes	15 (10.48)	1 (0.69)	16.64 (2.17-127.76)	<0.001*
No	128 (89.51)	142 (99.30)	1	-
History of preterm delivery				
Yes	29 (20.27)	12 (8.39)	2.78 (1.35-5.69)	0.004*
No	114 (79.72)	131 (91.60)	1	-
History of using steroid medications				
Yes	20 (13.98)	09 (6.29)	2.42 (1.06-5.52)	0.03*
No	123 (86.01)	134 (93.70)	1	-
History of congenital anomalies				
Yes	03 (2.09)	02 (01.39)	1.51 (0.25-9.18)	1.00
No	140 (97.90)	141 (98.60)	1	-
Maternal depression during pregnancy				
Yes (EPDS score ≥12)	47 (32.86)	23 (16.08)	2.55 (1.45-4.50)	0.001*
No (EPDS score <12)	96 (67.13)	120 (83.91)	1	-

Comparison of birth outcomes associated with GDM

Table 5 presents a comparison of birth outcomes (pregnancy, labor, and neonatal outcomes) between the two groups and the association of these outcomes with GDM, estimated using ORs.

**Table 5:** Association between birth outcomes and gestational diabetes mellitus (GDM) (n = 286). *Statistically significant (*P* < 0.05). Fisher's exact *P*-values were computed for small or imbalanced cell counts. Firth's penalized logistic regression was used for outcomes with zero cell counts. GDM, gestational diabetes mellitus; OR, odds ratio; CI, confidence interval

Variables	GDM (*n* = 143), *n* (%)	Non-GDM (*n* = 143), *n* (%)	OR (95% CI)	*P*-value
Pregnancy outcome				
Oligohydramnios				
Yes	05 (3.50)	01 (0.70)	5.14 (0.59-44.60)	0.13
No	138 (96.50)	142 (99.30)	1	-
Polyhydramnios				
Yes	04 (2.80)	05 (3.50)	0.79 (0.20-3.02)	0.73
No	139 (97.20)	138 (96.50)	1	-
Pre-eclampsia				
Yes	06 (4.20)	05 (3.50)	1.21 (0.36-4.05)	0.70
No	137 (95.80)	138 (96.50)	1	-
Labor outcome				
Mode of delivery				
Normal vaginal delivery	44 (30.76)	80 (55.90)	1	
Cesarean section	98 (68.53)	61 (42.65)	2.92 (1.79-4.76)	<0.001*
Instrumental delivery	01 (0.71)	02 (1.40)		
Premature rupture of membrane (PROM)				
Yes	15 (10.50)	7 (04.90)	2.28 (0.89-5.76)	0.08
No	128 (89.50)	136 (95.10)	1	-
Preterm delivery				
Yes	65 (45.45)	43 (30.07)	1.94 (1.19-3.15)	0.007*
No	78 (54.55)	100 (69.93)	1	-
Induction of labor				
Yes	79 (55.24)	33 (23.07)	4.11 (2.47-6.85)	<0.001*
No	64 (44.75)	110 (76.92)	1	-
Neonatal outcome				
Neonatal Intensive Care Unit (NICU) admission				
Yes	115 (80.42)	60 (41.95)	5.68 (3.34-9.64)	0.001*
No	28 (19.58)	83 (58.04)	1	
Neonatal hypoglycemia				
Yes	33 (23.07)	3 (2.10)	14.00 (4.18-46.85)	0.001*
No	110 (76.93)	140 (97.90)	1	-
Neonatal hyperbilirubinemia				
Yes	13 (09.09)	2 (1.40)	7.05 (1.56-31.84)	0.01*
No	130 (90.91)	141 (98.60)	1	-
Macrosomia (corrected with Firth regression)				
Yes	13 (9.09)	0 (0)	32.97 (4.30-4235.35)	0.01*
No	130 (90.91)	143 (100)	1	-
Intrauterine growth retardation (IUGR)				
Yes	15 (10.49)	7 (4.90)	2.27 (0.89-5.76)	0.08
No	128 (89.51)	136 (95.10)	1	-
Large for gestational age (LGA)				
Yes	9 (6.30)	16 (11.19)	0.53 (0.22-1.24)	0.14
No	134 (93.70)	127 (88.81)	1	-

In relation to intrapartum/labor outcomes, women with GDM had significantly higher odds of undergoing cesarean delivery (OR = 2.92; 95% CI: 1.79-4.76; *P* < 0.001) and preterm delivery (OR = 1.94; 95% CI: 1.19-3.15; *P* = 0.007) than those without GDM. The need for induction of labor was significantly higher among women with GDM (55.24% vs. 23.07%), with significantly higher odds (OR = 4.11, 95% CI: 2.47-6.85; *P* < 0.001) compared with non-GDM women.

With respect to neonatal outcomes, neonatal hypoglycemia, neonatal hyperbilirubinemia, macrosomia, and NICU admission were observed more frequently among neonates born to mothers with GDM than among those born to non-GDM mothers. Mothers with GDM had significantly higher odds of neonatal hyperbilirubinemia (OR = 7.05; 95% CI: 1.56-31.84; *P* = 0.01), neonatal hypoglycemia (OR = 14.00; 95% CI: 4.18-46.85; *P* = 0.001), giving birth to a macrosomia baby (OR = 32.97; 95% CI: 4.30-4235.35; *P* = 0.01), and being admitted to the NICU (OR = 5.68; 95% CI: 3.34-9.64; *P* = 0.001). Although higher odds were observed for neonatal hypoglycemia and macrosomia, the wide CIs suggest limited precision of the estimates, possibly due to the small number of outcome events.

## Discussion

In the present study, the point prevalence of GDM was 9.9% among 3,108 pregnant women enrolled over a six-month period at public maternity healthcare centers in Khurdha district, Odisha. This finding is comparable to a previous study from the same region that reported a GDM prevalence of 9.89% [12]. The similarity in prevalence may be attributed to comparable geographical settings, dietary patterns, socioeconomic conditions, and lifestyle characteristics.

This study further examined the association of GDM with sociodemographic, socioeconomic, lifestyle, and obstetric characteristics. Previous research has consistently identified maternal age, obesity, family history of diabetes, previous GDM, parity, history of abortion, preterm birth, congenital anomalies, and previous delivery of a macrosomic infant as important risk factors for GDM [7-11,18]. Although several studies have demonstrated that advancing maternal age and delayed menarche are associated with an increased risk of GDM [25,26], our findings differed in that early menarche (≤12 years) was associated with a reduced risk of GDM. This observation is supported by a large cohort study conducted in the United States, which reported that women with menarche before 11 years of age had a higher risk of developing GDM during their first pregnancy compared with those who attained menarche between 11 and 15 years. Similarly, a recent meta-analysis found that women experiencing menarche at ≤11 years had a significantly greater risk of GDM than those with menarche at ≥12 years [25,26].

Among lifestyle-related factors, obesity and low physical activity were significantly associated with GDM in the present study. These findings are consistent with previous reports indicating that unhealthy dietary habits, sedentary behavior, and poor sleep quality contribute to metabolic alterations during pregnancy, thereby increasing the risk of GDM [27-29]. In contrast, some studies have not observed a significant association between obesity and GDM [10,13]. Nevertheless, maintaining a healthy diet and engaging in regular physical activity remain the most effective strategies for preventing excessive gestational weight gain.

Genetic susceptibility has been suggested as a factor contributing to the development of GDM. In the current study, both a family history of diabetes and a previous history of diabetes were significant predictors of GDM. These findings are in agreement with earlier studies showing that a family history of diabetes, particularly among first-degree relatives, as well as prediabetes, substantially increases the likelihood of developing GDM [7,30]. Additionally, obstetric factors including a previous history of GDM, primiparity, previous delivery of a macrosomic infant, history of preterm birth, and prior use of steroid medications were significantly associated with GDM, corroborating findings reported in earlier studies [14,15,24]. Maternal depression during pregnancy was found to be significantly associated with an increased risk of GDM in the present study. This result is consistent with evidence from a recent meta-analysis demonstrating that depression during pregnancy is linked to a significantly higher risk of developing GDM [31].

Regarding pregnancy outcomes, previous studies have reported that untreated or inadequately managed GDM is associated with increased risks of fetal macrosomia, neonatal hypoglycemia, neonatal hyperbilirubinemia, stillbirth, preterm birth, and maternal complications such as preeclampsia, polyhydramnios, cesarean delivery, and instrumental birth [20,21,24,32]. Similarly, our findings demonstrated that women with GDM had a higher frequency of macrosomia, neonatal hypoglycemia, neonatal hyperbilirubinemia, cesarean delivery, preterm birth, and neonatal intensive care unit (NICU) admission compared with women without GDM. However, no significant associations were observed between GDM and stillbirth, intrauterine growth restriction (IUGR), or large-for-gestational-age infants. These findings are consistent with those of a population-based cohort study, which suggested that timely diagnosis and appropriate management of GDM can substantially reduce the risk of adverse maternal and neonatal outcomes, including the need for cesarean delivery [32].

Differences in the severity of hyperglycemia, glycemic control, treatment received, and other clinical characteristics may have contributed to the observed differences in outcomes. As these factors were not consistently available or adequately controlled for in the present analysis, residual confounding cannot be excluded. Moreover, referral patterns and case mix are likely to have influenced both prevalence estimates and observed outcomes in this study. As the study setting functions as a referral center, it may have received a higher proportion of GDM cases, potentially influencing prevalence and adverse outcome rates. Additionally, variations in healthcare availability, including diagnostic and management services, may affect outcome measures and limit direct comparison with population-based studies.

Study limitation

The researchers reported some of the study's limitations: First, it is a local study conducted at selected maternity hospitals in Khurdha district, Odisha, so the results may not generalize to populations in other settings in India. Moreover, the current study only used one screening technique to identify GDM cases and lacked data on the quality of care GDM mothers received and a comparison of that risk with the pregnancy outcome. However, the matching strategy by GA limited the ability to examine GA as a risk factor and may restrict the generalizability of the findings to populations with wider gestational ranges. Recall bias might have been introduced due to reliance on self-reported information and historical data from study participants, but the accuracy of some data was assessed by reviewing their antenatal records. Courtesy and social disability bias were present, as the researchers were in clinical uniform.

## Conclusions

According to the study's findings, lifestyle factors such as preconception obesity, sedentary lifestyle, history of diabetes, family history of diabetes, and domestic violence were found to be significant risk factors for GDM. Obstetrical factors such as early menarche (<13 years), primiparity, previous history of GDM, prior history of macrosomia, preterm delivery, use of steroid medications, and antepartum depression were all associated with GDM in subsequent pregnancies. It was also found that adverse intrapartum and neonatal outcomes, particularly cesarean delivery, preterm delivery, induction of labor, NICU admission, neonatal hypoglycemia, hyperbilirubinemia, and macrosomia, were significantly more frequent among women with GDM compared with non-GDM mothers. One of the greatest health issues in the world today is GDM, which calls for efficient preventive and control measures. To reduce long- and short-term adverse outcomes for mothers and offspring, early detection and effective management of GDM are also required, especially in low- and middle-income countries such as India. Therefore, preconception screening and counseling may be considered as part of the continuum of care within routine maternal health services, together with appropriate, need-based management, to facilitate early identification and modification of risk factors and potentially reduce adverse birth outcomes associated with GDM.
